# A pupil size response model to assess fear learning

**DOI:** 10.1111/psyp.12801

**Published:** 2016-12-07

**Authors:** Christoph W. Korn, Matthias Staib, Athina Tzovara, Giuseppe Castegnetti, Dominik R. Bach

**Affiliations:** ^1^ Department of Psychiatry, Psychotherapy, and PsychosomaticsUniversity of ZurichZurichSwitzerland; ^2^ Neuroscience Center ZurichUniversity of ZurichZurichSwitzerland; ^3^ Wellcome Trust Centre for NeuroimagingUniversity College LondonLondonUK

**Keywords:** General linear model, Response functions, Auditory, Visual, Somatosensory

## Abstract

During fear conditioning, pupil size responses dissociate between conditioned stimuli that are contingently paired (CS+) with an aversive unconditioned stimulus, and those that are unpaired (CS‐). Current approaches to assess fear learning from pupil responses rely on ad hoc specifications. Here, we sought to develop a psychophysiological model (PsPM) in which pupil responses are characterized by response functions within the framework of a linear time‐invariant system. This PsPM can be written as a general linear model, which is inverted to yield amplitude estimates of the eliciting process in the central nervous system. We first characterized fear‐conditioned pupil size responses based on an experiment with auditory CS. PsPM‐based parameter estimates distinguished CS+/CS‐ better than, or on par with, two commonly used methods (peak scoring, area under the curve). We validated this PsPM in four independent experiments with auditory, visual, and somatosensory CS, as well as short (3.5 s) and medium (6 s) CS/US intervals. Overall, the new PsPM provided equal or decisively better differentiation of CS+/CS‐ than the two alternative methods and was never decisively worse. We further compared pupil responses with concurrently measured skin conductance and heart period responses. Finally, we used our previously developed luminance‐related pupil responses to infer the timing of the likely neural input into the pupillary system. Overall, we establish a new PsPM to assess fear conditioning based on pupil responses. The model has a potential to provide higher statistical sensitivity, can be applied to other conditioning paradigms in humans, and may be easily extended to nonhuman mammals.

Fear conditioning paradigms rank among the most fundamental and widespread experimental procedures to elucidate the neurophysiology of aversive learning across different species (Maren, 2001). Discriminant delay fear conditioning involves presenting initially neutral, conditioned stimuli (CS), in various modalities, one of which (CS+) coterminates with an aversive unconditioned stimulus (US), such as a mild electric stimulation, while the other (CS‐) is always presented alone.

Fear conditioning is often probed by measuring autonomic responses. Several studies have demonstrated differential pupil size responses to CS+/CS‐ in cats (Oleson, Ashe, & Weinberger, 1975; Oleson, Westenberg, & Weinberger, 1972) and humans (Greenberg, Carlson, Cha, Hajcak, & Mujica‐Parodi, 2013; Hermans et al., 2016; Kluge et al., 2011; Pollak et al., 2010; Reinhard & Lachnit, 2002; Reinhard, Lachnit, & König, 2006; Visser et al., 2016; Visser, Kunze, Westhoff, Scholte, & Kindt, 2015; Visser, Scholte, Beemsterboer, & Kindt, 2013). This resonates with a rich literature showing that pupil size is influenced by a wide range of neuropsychological processes in humans (Granholm & Steinhauer, 2004) such as perception of various arousing stimuli (Bayer, Sommer, & Schacht, 2011; Bradley, Miccoli, Escrig, & Lang, 2008; Hermans, Henckens, Roelofs, & Fernández, 2013; Korn & Bach, 2016; Prehn et al., 2013; Preller et al., 2014; Võ et al., 2008). However, there is a lack of universally accepted quantitative methods for analyzing pupil responses, and the approaches used at present are rather inhomogeneous and unspecified. They typically rely on methods such as peak scoring or calculating the area under the curve (AUC) within ad hoc defined time windows.

Here, we sought to formalize extant knowledge on pupil size responses to CS+ and CS‐ in a psychophysiological model (PsPM). This affords a more principled approach to estimating the underlying fear learning process (Bach & Friston, 2013). Fear learning, in this approach, is quantified by the amplitude of a cognitive input into the pupil system. The aim of the current study was to develop a method that provides a minimum variance estimate of this cognitive input. In order to evaluate the method and compare it with previous approaches to the quantification of fear learning, we relied on simple fear conditioning experiments in which it can be assumed a priori that a CS/US association is established and pupil responses to CS+ or to CS‐ can be assumed to differ on average. Although in these validation experiments only two conditions need to be distinguished (a discrimination problem), the goal of the method is to provide minimum‐variance estimates of fear learning also in situations in which it may vary across more than just two levels, for example, to provide trial‐by‐trial estimates of fear learning or to assess parametric manipulations of fear memory. Of course, since pupil size reflects many other psychological processes, too (Korn & Bach, 2016), a specific inference on fear learning rests on a suitable experimental design in which conditions only differ on this dimension.

As in a previous study (Korn & Bach, 2016), we conceptualized pupil size responses as output of a linear time‐invariant system that receives cognitive inputs at the onset of the CS. The amplitude of these neural inputs supposedly depends on a fear learning process. This linear time‐invariant system is unambiguously characterized by its impulse response function. Under these assumptions, the amplitude of putative inputs can be inferred from observed data by inverting a general linear model (GLM). For such a GLM, the design matrix is formed by convolving event onsets with the impulse response function with unit amplitude. This approach is commonplace in the analysis of neuroimaging data (Friston, 2005; Friston, Jezzard, & Turner, 1994), and has more recently been extended in the form of PsPM to skin conductance responses (SCR; Bach, Flandin, Friston, & Dolan, 2009), heart period responses (Paulus, Castegnetti, & Bach, 2016), and respiratory responses (Bach, Gerster, Tzovara, & Castegnetti, 2016). A similar approach has been used for pupil size measurements related to detecting auditory events (Knapen et al., 2016) and perceptual decision making (de Gee, Knapen, & Donner, 2014). Overall, estimating neural processes via model‐based approaches tends to improve the signal‐to‐noise ratio (Bach & Friston, 2013). For example, model‐based analysis of SCR (Staib, Castegnetti, & Bach, 2015), heart period responses (Castegnetti et al., 2016), respiration responses (Castegnetti, Tzovara, Staib, Gerster, & Bach, in press), and possibly also startle eyeblink responses (Khemka, Tzovara, Gerster, Quednow, & Bach, 2016) provides better discrimination of CS+/CS‐ responses than peak scoring measures in fear conditioning paradigms.

For some data types (e.g., skin conductance), responses to CS+ and CS‐ look very similar and only differ by their amplitude (Boucsein, 1992). For others, such as heart period, responses appear to be composed of several components: one reflecting a response to the CS, which is the same for CS+ and CS‐, and an added component with different time course for the CS+ (Castegnetti et al., 2016). Crucially, in this study, it appeared that the common heart period response to the CS differs across experiments and may depend on specific perceptual characteristics that do not necessarily generalize across studies. However, we found that by only modeling the CS+‐related component, CS+ and CS‐ could be reliably distinguished, such that there is no need to model common CS responses (Castegnetti et al., 2016). Indeed, additionally modeling such responses would influence parameter estimates for CS‐ and CS+ equally, such that their contrast would be unchanged. This is the approach that we pursue here for pupil size responses.

Thus, our first aim was to develop a model that describes fear‐conditioned pupil size responses. Such a model would provide a useful methodological tool to enhance power when analyzing fear conditioning experiments. To test wide generalizability, we validated the model on three experiments with CS from different sensory modalities (visual, auditory, and somatosensory) and one experiment with a longer presentation of auditory CS. Additionally, we compared the newly developed pupil model to SCR and heart period models with respect to their discrimination of CS+ and CS‐.

Crucially, the system modeled by our response functions probably collapses an effector organ system together with parts of the peripheral and central neural system. The pupillary system offers the advantage that the system is partly known. Changes in luminance elicit pupillary responses via a well‐characterized neural circuit (McDougal & Gamlin, 2008) and share a common final (neural and muscular) pathway with pupil responses elicited by cognitive processes (Korn & Bach, 2016). This allowed us to build on our previous model for such luminance‐related pupil responses (Korn & Bach, 2016), which corresponds to the common parts of the system and estimate the temporal evolution of inputs from higher nodes in the central nervous system into this pupillary system during fear conditioning. The plausibility of this result demonstrates consistency between the current and our previous model.

## Method

### Participants

We recruited participants for five independent fear conditioning experiments from the general and student population (see Table 1 for details). Participants reported to be healthy and drug free. All five samples were independent except for two persons: one participated in Experiment 1 and 3 and one in Experiment 1 and 4. The a priori criterion for excluding participants from analysis was more than 35% of missing data points (see Data Preprocessing). Participants received monetary compensation. The study, including the form of taking written informed consent, was conducted in accordance with the Declaration of Helsinki and was approved by the governmental research ethics committee (Kantonale Ethikkommission Zürich).

**Table 1:** Overview of Tasks and Participants

Experiment	*n* initial sample	Age in years (mean ± *SD*)	*n* female	*n* final sample
Experiment 1: Auditory CS (40 CS+US+, 40 CS+US‐, 80 CS‐)	22	26.4 ± 5.2	15	19
Experiment 2: Auditory CS (48 CS+US+, 48 CS+US‐, 96 CS‐)	20	23.1 ± 3.0	10	12
Experiment 3: Visual CS (24 CS+US+, 24 CS+US‐, 48 CS‐, 96 stimuli in nonreinforced context)	20	27.7 ± 5.6	15	17
Experiment 4: Somatosensory CS (24 CS+US+, 24 CS+US‐, 48 CS‐)	18	24.4 ± 4.4	7	18
Experiment 5: Long auditory CS (20 CS+US+, 20 CS+US‐, 40 CS‐)	21	22.6 ± 3.3	13	15

### Psychophysiological Recording and Delivery of Unconditioned Stimuli

Testing was performed in a dark, soundproof chamber (with background illumination provided by the monitor; see experimental setup for specific luminance values at participants' eyes). Participants' heads were positioned on a chin rest in front of the monitor (Dell P2012H, 20” set to an aspect ratio of 5:4, 60 Hz refresh rate). Head‐to‐monitor distance was 70 cm in Experiment 1 and 55 cm otherwise. Pupil diameters and gaze direction for both eyes were recorded with an EyeLink 1000 System (SR Research, Ottawa, ON, Canada) at a sampling rate of 500 Hz unless otherwise indicated. We used the 9‐point calibration implemented in the EyeLink 1000 software for calibrating gaze direction.

Skin conductance was recorded from the thenar/hypothenar of the nondominant hand with two 8‐mm disk Ag/AgCl cup electrodes (EL258, Biopac Systems Inc., Goleta, CA) and 0.5% NaCl gel (GEL101, Biopac; Hygge & Hugdahl, 1985). The SC signal was measured with an SCR coupler/amplifier (V71‐23, Coulbourn Instruments) and digitized at 200 Hz, using a Dataq card (DI‐149, Dataq Inc., Akron, OH) and recorded with Windaq (Dataq Inc.) software.

In Experiment 1 and 2, electrocardiogram (ECG) was recorded with four 45‐mm, pregelled Ag/AgCl adhesive electrodes attached to the four limbs. The experimenter visually identified the lead (I, II, III) or the augmented lead (aVR, aVL, aVF) configuration that displayed the highest R spike, and only recorded this configuration. Data were preamplified and 50 Hz notch‐filtered with a Coulbourn isolated five‐lead amplifier (LabLinc V75‐11, Coulbourn Instruments, Whitehall, PA), digitized at 1000 Hz.

The US consisted of a train of electric square pulses delivered with a constant current stimulator (Digitimer DS7A, Digitimer, Welwyn Garden City, UK) on participants' dominant forearm through a pin‐cathode/ring‐anode configuration. The experimenter set the current such that perceived shock intensity was just below the pain threshold. The pain threshold was estimated in a procedure consisting of two phases. First, the experimenter increased the intensity from an unperceivable to a painful level. The latter was then used as the upper threshold in the second phase. Participants rated the perceived intensities of 14 stimuli of different intensities on a scale from 0% (*no sensation*) to 100% (*very unpleasant*). For the experiment, we used an intensity that participants would have rated as 85% (based on a linear interpolation of the 14 subjective ratings).

### Experimental Setup

#### Common settings–timing

All tasks used a delay fear conditioning procedure. In the first four experiments, CS presentations lasted 4 s. In the fifth experiment, CS were presented for 6.5 s. Half of the occurrences of the CS+ were reinforced with a US (CS+US+ trials), and half of them were not reinforced (CS+US‐ trials). In CS+US+ trials, US onset was at 0.5 s before CS+ offset. CS‐ were never paired with a US (CS‐ trials). The temporal order of the different trial types was randomized. The intertrial interval was randomly determined to be 7, 9, or 11 s in the first four experiments and 11, 15, or 17s in the fifth experiment. Assignment of stimuli to either CS+ or CS‐ was counterbalanced across participants.

#### Common settings–behavioral responses

To ensure that participants remained awake throughout the experiment, they were instructed to indicate the difference between the physical properties of the CS stimuli (i.e., we did not ask participants to indicate whether the stimulus was CS+ or CS‐; instead, we asked them to indicate, for example, whether the tone was low or high pitched; see below). They had to press one of two keys for each of the CS types. Keys were counterbalanced across participants.

#### Data use

For model development, we used data from Experiment 1, which were collected specifically for the current report. For model validation, we used data from Experiment 2 to 4, which were collected in the context of other studies. Pupil data from these experiments has not been reported previously. Experiment 5 was specifically collected for this study and was used to test whether the model can be extended to setups with longer CS presentation. The data sets of the five experiments will be made available in an online repository (accessible via http://pspm.sourceforge.net).

#### Software

All experiments were programmed in MATLAB using the Cogent 2000 toolbox (www.vislab.ucl.ac.uk) for Experiment 1, 2, and 5 and psychtoolbox (http://psychtoolbox.org) for Experiment 3 and 4.

#### Experiment 1–Simple auditory CS

CS consisted of two sine tones with constant frequency (220 Hz or 440 Hz, 50‐ms onset and offset ramp). Sound stimuli were created in MATLAB and converted to sound files with the inbuilt *wavwrite* function. Sounds were played with Cogent 2000 and delivered via headphones at approximately 60 dB (HD 518, Sennheiser, Wendemark‐Wennebostel, Germany). Assignment of low‐ and high‐pitched sounds to CS+ and CS‐ was counterbalanced across participants. During the entire task, a white fixation cross (height/width 1.67° visual angle) was presented on a gray background (72.7 cd/m^2^). In total, there were 40 CS+US+, 40 CS+US‐, and 80 CS‐ trials in two sessions, which were separated by a brief self‐paced break.

#### Experiment 2–Simple and complex auditory CS

SCR and ECG data from this experiment were published previously (Castegnetti et al., 2016; Staib et al., 2015). Two sets of CS+/CS‐ were used (simple stimuli: sine tones with a constant frequency of 400 or 800 Hz over the entire 4‐s interval; complex stimuli: a train of four frequency modulated sounds of 1 s each, which were rising from 400 to 800 Hz or falling from 800 to 400 Hz). Stimuli were delivered at about 68 dB with headphones (HD518, Sennheiser). Assignment of the auditory stimuli to CS+ and CS‐ was counterbalanced across participants. Trials were presented in 16 miniblocks of 12 trials each, which were separated by brief self‐paced breaks. Simple or complex stimuli were presented in alternating miniblocks. For all analyses reported here, we pooled responses to simple and complex stimuli since analyses of the SCR data did not reveal an interaction between fear learning and complexity. In 25% of all trials, a brief startle sound was presented. To avoid potential biases in the pupillary responses due to startle sounds, these trials were excluded from all analyses presented here. A white fixation cross (height/width 0.42° visual angle) was presented on a gray background (77.1 cd/m^2^) throughout the task. Participants had to indicate the physical property of the CS (i.e., whether simple CS were low or high pitched, or whether complex CS were rising or falling). If participants failed to respond or responded incorrectly, the fixation cross turned red. To avoid any possible contamination of pupil traces with luminance changes and/or reactions to feedback about missing or wrong performance, these trials were excluded from all analyses (mean percentages ± standard deviations of included trials disregarding the startle trials: CS+US+ 0.93 ± 0.10; CS+US‐ 0.97 ± 0.06; CS‐ 0.96 ± 0.06).

#### Experiment 3–Visual CS

Four sets of CS+/CS‐ were used. Simple CS consisted of Gabor patches rotated to the left or to the right; complex CS consisted of plaids created from two Gabor patches that were overlaid on each other with a 230° angle, rotated to the left or to the right. Diameter of the Gabor patches was 13.79° visual angle, and the height/width of the fixation cross in intertrial intervals was 0.83° visual angle. Assignment of the visual stimuli to CS+ and CS‐ was counterbalanced across participants. The experiment was split into 16 miniblocks of 12 trials each. In each miniblock, either simple or complex CS were presented. Eight miniblocks used complex and simple CS (reinforced). In the remaining eight miniblocks, participants were instructed that no US would occur (nonreinforced). These nonreinforced miniblocks allowed us to measure the “pure” pupil responses to the sensory features of the visual stimuli. Odd‐numbered miniblocks were reinforced and even‐numbered miniblocks were not reinforced. Participants were explicitly informed whether reinforcement would occur within a miniblock type or not. This was signaled by different background colors (yellow: 116.9 cd/m^2^; light purple: 101.1 cd/m^2^), which were counterbalanced across participants. Importantly, they were not informed which of the two CS stimuli presented in a reinforced miniblock would be associated with the US. That is, they had to learn the CS‐US contingencies just as in the other experiments. We report mean responses to all stimuli in the nonreinforced blocks; otherwise, data from these blocks were not analyzed. Following the same rationale as Experiment 2, we pooled data for simple and complex stimuli and excluded trials in which participants did not correctly indicate the physical properties of the CS (mean percentages ± standard deviations of included trials: CS+US+ 0.81 ± 0.15; CS+US‐ 0.82 ± 0.19; CS‐ 0.85 ± 0.17). In 10 participants, the sampling rate of the pupil recordings was 1000 Hz. Data from these participants were downsampled to 500 Hz.

#### Experiment 4–Somatosensory CS

Two sets of somatosensory CS+/CS‐ were applied to the intermediate phalanges of the index and middle fingers of the nondominant hand using one constant current stimulator for each finger (Digitimer DS7A, Digitimer; simple stimuli: stimulations to either index or middle finger; complex stimuli: stimulations of different temporal structure to both index and middle fingers). CS intensity was set to a perceivable but not unpleasant level. To ensure that participants were able to discriminate the somatosensory stimuli, we included a training session without any reinforcement before the actual fear conditioning experiment. To reduce generalization from this initial nonreinforcement context, the screen background color was changed from yellow (98.0 cd/m^2^) to light purple (97.3 cd/m^2^) or vice versa (counterbalanced across subjects). Participants were asked to fixate a white fixation cross (height/width 1.15° visual angle). Again, we pooled data for simple and complex stimuli and excluded trials in which participants did not correctly indicate the physical properties of the CS (mean percentages ± standard deviations of included trials: CS+US+ 0.94 ± 0.08; CS+US‐ 0.95 ± 0.07; CS‐ 0.93 ± 0.09).

#### Experiment 5–Long simple auditory CS

All settings were the same as in Experiment 1 except for the timing and the trial numbers. CS duration was 6.5 s (and not 4 s), and thus the US+ lasted from 6 to 6.5 s (and not from 3.5 to 4 s) after CS onset. There were 20 CS+US+, 20 CS+US‐, and 40 CS‐ trials in two sessions, which were separated by a brief self‐paced break.

### Data Preprocessing

The EyeLink 1000 System uses an online parsing algorithm to detect saccades and fixations. All subsequent analyses were performed in MATLAB (Version R2013a, Math Works, Natick MA) on the basis of the routines reported in our previous article (Korn & Bach, 2016). Time series were analyzed from the beginning of the first stimulus presentation until 15 s after the last event.

Pupil measurements obtained from a video‐based eye tracker depend on the gaze angle (Hayes & Petrov, 2015). Therefore, participants were asked to fixate a central fixation cross in all experiments. No explicit free viewing or blinking periods were included in the tasks. If fixation was not maintained, trials were not aborted or repeated (because this would have altered the contingencies in the fear conditioning procedures). Instead, we excluded missing data points from the analyses as detailed in the following paragraph. We did not filter the pupil time series because in our previous analyses (Korn & Bach, 2016) filtering did not affect our results (or made them less interpretable).

All data points for which *x*‐ or *y*‐gaze positions exceeded an a priori threshold of ± 5° visual angle were treated as missing data points. For the data reported in our previous article (Korn & Bach, 2016), including or excluding data points for which fixation was broken resulted in quantitatively very similar results. Overall, missing data points could potentially result from exceeding the gaze angle threshold, blinking, or brief head movements. Since we excluded missing data points, we did not distinguish between the different reasons for missing data points (i.e., we did not track head movements). Specifically, all missing data points were linearly interpolated for preprocessing but subsequently excluded before model inversion. Within each participant, we analyzed the pupil (left or right) for which more data points were available. If more than 35% of the data points were missing, participants were excluded from analyses, similar to our previous work (Korn & Bach, 2016).

Time series were *z*‐scored (by subtracting the mean and dividing by the standard deviation) within each session and participant to account for between‐subjects variance in overall pupil size. Preprocessing routines and the final model will be implemented in the open‐source MATLAB toolbox PsPM, which is freely available under the GNU General Public License and obtainable from http://pspm.sourceforge.net

### Mean Responses and Empirical Response Functions

To obtain a mean response, we extracted data segments following trial onset, and averaged these segments first within and then across participants. The segments were 10 s long. For illustration and for the development of the response functions, grand mean responses were baseline corrected by subtracting the respective first data point from the entire length of the segments.

We took a data‐driven approach to develop a linear time‐invariant system that models the difference of the anticipatory responses elicited by CS+ versus CS‐. A linear time‐invariant system is unambiguously characterized by its response function. To derive such a response function empirically, we used the difference in the grand means between responses to CS+US‐ and CS‐ in the data set of Experiment 1. In this phenomenological approach, neither the exact analytical form of the response function nor its parameters were intended to reflect biophysical reality. A gamma probability density function offered an analytical description that provided a good approximation to the empirical data:

$$d\left(t\right)=c\frac{\;({t-t_{0})}^{k-1}\;e^{\frac{-(t-t_{0})}{\theta }}}{\theta^{k}\Gamma (k)},$$

where *d* is the *z*‐scored steady‐state pupil diameter, *t* is time, *t*_0_ is the onset latency, and Γ is the gamma function, and *t*_0,_ *k*, *θ*, and *c* are free parameters. Parameters were estimated using ordinary least squares minimization and a Nelder‐Mead simplex search algorithm as implemented in the MATLAB function *fminsearch*.

### Model Inversion

This function can then be convolved with a time series of unity inputs, which specify the occurrences of CS, to obtain a predicted time series of pupil responses under the given model with unity amplitude. This predicted time series is compared to the observed data time series of pupil size measurements by inverting a GLM. The amplitudes of the putative inputs into the pupil are the parameter estimates of the GLM. Such a GLM can be written as

Y=Xβ+ɛ,

where *Y* is the measured pupil data time series, *X* is the design matrix (which contains the time series of the different kinds of inputs representing the experimental design, convolved with the components of the response functions), β is a vector of amplitude parameters, and ɛ is normally distributed noise (Friston, 2005; Friston et al., 1994). Specifically, we inverted GLMs separately for each participant to infer parameter estimates for the participant‐specific amplitudes of the input into the pupillary system. We separately modeled CS+US‐ and CS‐ responses, such that the resulting design matrix included three regressors, one of them specifying the intercept. Furthermore, we implemented additional GLMs that use the derivative of the response function as an additional basis function (resulting in a design matrix with five regressors). All GLMs were inverted using a maximum likelihood method implemented in the MATLAB function *pinv*. Participant‐specific amplitudes were then determined as parameters of the corresponding design matrix column (one‐component response function). In the case of two‐component response functions, we reconstructed the estimated response by multiplying the basis set with the response estimates. We then computed the maximal (signed) excursion of the reconstructed response for each CS type to quantify the amplitude, and this summarizes the first‐level model into one parameter per condition (Bach, Friston, & Dolan, 2013). As described above, time periods with missing data were removed before model inversion.

### Model Comparison and Validation

The aim of the current report is to find the procedure that best distinguishes pupil responses to CS+ and CS‐, where the temporal occurrences of CS+ and CS‐ are known a priori. For this purpose, we compared our approach with two classical methods, namely, peak scoring and taking the AUC. Our model‐based approach is based on a finite linear time invariant system that thus requires that response functions “return to baseline” (i.e., the beginning and the ending of the response functions need to take the same value). To fulfill this, we based the estimation of the pupil response functions on a time window of 10 s. Therefore, we also based peak scoring and calculating the AUC on a time window of 10 s after CS onset. This mitigates a potential criticism that a model‐based approach is more sensitive than peak scoring only because it is based on a larger data window. Nevertheless, we also verified our main results by performing peak scoring and calculating the AUC in windows of the lengths of the CS duration.

We calculated the signed maximum for peak scoring and the signed AUC in each CS+US‐ and CS‐ trial and averaged these within these two conditions (i.e., CS+US‐ and CS‐) within participants. For the PsPMs, we used the participant‐specific amplitudes for the two CS types as estimated from the inverted GLMs (i.e., the maximal signed excursion of the reconstructed response for each CS type; see previous section).

To compute the validity for each method, the participant‐specific values for the two CS conditions (i.e., means of peaks, means of AUCs, or estimated amplitudes) were then inserted into a regression model. In this model, known event types (CS+ or CS‐) define the dependent variable, and estimated response amplitudes to CS+US‐ and CS‐ for each participant serve as independent variable, complemented by regressors for subject‐specific intercepts. The residual sum of squares (RSS) from this regression model was then transformed into Akaike Information Criterion (AIC), which specifies the evidence for a model in which CS+ and CS‐ estimates are drawn from distributions with different means, rather than the same mean. This is analogous to a paired *t* test, and we provide *t* and *p* values for the paired sample *t* test across participants. To compute AIC, we used the following formula (Burnham & Anderson, 2004):

$$AIC=n\;\log\left(\frac{RSS}{n}\right)+2(r+1),$$

where *n* is the number of observations (i.e., two observations, CS+US‐ and CS‐, per participant in our case) and *r* is the number of regressors in the regression model. The values for *n* and *r* were the same for all methods such that differences in AIC between methods are identical to differences in negative log likelihood, or Bayesian information criterion. This is because results from all first‐level models, including those containing more complex basis functions, were collapsed into one amplitude parameter per condition. AIC differences can be interpreted as log Bayes factors, where smaller AIC values indicate higher predictive validity (Bach & Friston, 2013; Castegnetti et al., 2016; Staib et al., 2015). An absolute AIC difference of more than 3 is often regarded as decisive, by analogy to a classic *p* value (Penny, Stephan, Mechelli, & Friston, 2004; Raftery, 1995): a classic *p* < .05 means that the probability of the data given the null model is smaller than 5%. An absolute AIC difference larger than 3 means that the probability of the inferior model given the data is smaller than 5% (since *p* < *e* ^−3^ ≈ .05).

We also report effect sizes of the CS+/CS‐ difference as Cohen's *d*, which relates to the *t* value of a paired *t* test by

$$d=\frac{t}{\sqrt{n}}.$$

We validated the pupil model derived from Experiment 1 in Experiment 2 to 4. For future applications, we wanted to base the most promising model on the maximum amount of data available to us and therefore derived an additional response function from the data of the first four experiments combined. We checked that the results obtained with that model were consistent with those of the model using the response function based on Experiment 1 only. For illustration, we also depict separate response functions for the three validation data sets of Experiment 2 to 4, but these were not used for analyzing the data.

### Analyses of SCR and Heart Period Data

To put our pupil size model into perspective, we sought to compare it to other psychophysiological measures. Previous reports have established PsPM approaches for SCR and ECG data that outperform classic peak‐scoring approaches in discriminating responses to CS+US‐ versus CS‐ (Castegnetti et al., 2016; Staib et al., 2015). We therefore used the model comparison procedure described above to test whether the three measurement modalities differ in their predictive validity. SCR and ECG data for Experiment 2 have been reported previously (Castegnetti et al., 2016; Staib et al., 2015). Data from the other experiments have not been reported. In Experiment 1, we recorded SCR and ECG data; in Experiment 3 and 4, we only recorded SCR data.

We used the default analyses procedures described previously and as implemented in PsPM 3.0. In brief, for analysis of the SCR, we used the default dynamical causal model (DCM; Bach, Daunizeau, Friston, & Dolan, 2010; Staib et al., 2015) with an anticipatory sympathetic arousal window corresponding to the CS/US interval. ECG data were converted to heart period time series, which were analyzed using the default GLM for fear‐conditioned heart period responses (Castegnetti et al., 2016). Again, the number of regressors *r* were the same for all methods such that differences in AIC between methods are identical.

### Modeling the Inputs into the Pupillary System Elicited by Fear Conditioning

On the basis of the luminance response model, established in our previous report (Korn & Bach, 2016), we estimated the inputs that elicit pupil size responses related to the anticipation of aversive stimuli (i.e., to CS+US‐ versus CS‐ responses) and to the aversive stimuli themselves (i.e., to CS+US+ versus CS+US‐ responses). For this, we assumed inputs in the form of gamma probability density functions with four free parameters (see Mean Responses and Empirical Response Functions). We fitted the convolution of the luminance‐related response function with the assumed input to the normalized pupil size response (using ordinary least squares minimization and a Nelder‐Mead simplex search algorithm).

## Results

### Model Development on the Basis of Pupillary Responses in Auditory Fear Conditioning

**Figure 1:**
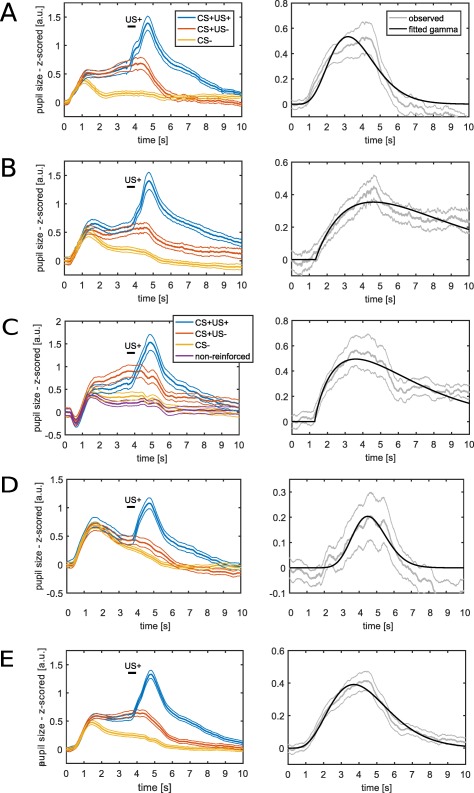
Mean pupil size responses (left) and response functions (right) fitted to the differences between CS+US‐ and CS‐. Thick lines represent mean responses and thin lines represent standard *SEM*. CS presentation lasted from 0 s to 4 s and US delivery occurred at 3.5 s after CS onset. In the right panels, fitted response functions are shown in black. These response functions describe the standard shape of the pupil size response to the difference between CS+US‐ and CS‐ and thus characterize the linear time‐invariant systems used to model the pupil time series. A: Experiment 1: Auditory fear conditioning with simple tones. This data set was used for model development. B–D: The presented response functions are depicted for illustration. B: Experiment 2—Auditory fear conditioning. C: Experiment 3—Visual fear conditioning. D: Experiment 4—Somatosensory fear conditioning. E: Average across all four experiments. Experiment 2–4 were used for model validation. The response function depicted in (E) was used for the analyses reported in Table 4.

Mean pupil size responses in Experiment 1 showed an influence of auditory fear conditioning on pupil size (Figure 1A). Both CS+ and CS‐ elicited an initial stimulus‐related dilation, which reached an initial peak around 1.1 s after CS onset. This dilation was sustained until around 4.5 s in CS+US‐ trials. In contrast, pupil size decreased in CS‐ trials to a lower level after the initial dilation. Thus, CS+ and CS‐ trials differed before US delivery. As expected, the US+ resulted in a rapid pupil dilation that peaked at around 1.1 s after US onset. At around 6 s after CS offset, pupil size reached baseline levels. Since the intertrial interval randomly lasted 7, 9, or 11 s, this makes it very unlikely that pupil responses in a given trial were influenced by pupil responses in preceding trials.

**Figure 2:**
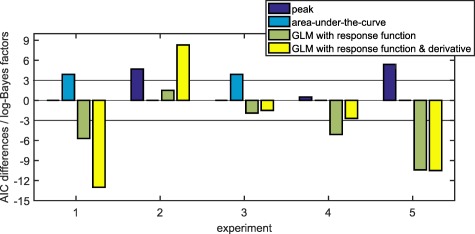
Graphical illustration of the differences in predictive validity (i.e., AIC) between the different methods (see Table 3 for numerical values of the same data and for the corresponding *t*, *p*, and *d* values). For each experiment, the difference in AIC was calculated with respect to the best non‐PsPM method (peak scoring for Experiment 1 and 3 and AUC for the other experiments). The horizontal line represents the decision thresholds of ± 3. In Experiment 1, 4, and 5, PsPMs outperformed peak scoring and calculating the AUC. In Experiment 2 and 3, PsPMs did not differ decisively from peak scoring or calculating the AUC.

For model development, we fitted a gamma probability density function to the difference between CS+US‐ and CS‐ trials (Figure 1A, see Table 2 for parameter values). We then used this as response function in a GLM. This model distinguished CS+ and CS‐ better (i.e., had better predictive validity) than two commonly used methods (i.e., comparing the peaks or the AUC, between conditions; see indicators Figure 2, Table 3). Including the derivative of this response function improved predictive validity further in Experiment 1.

**Table 2:** Parameters of Response Functions for the Difference Between CS+US‐ and CS‐

Experiment	*k*	*θ* [s^−1^]	*c*	*t*_0_ [s]
Experiment 1: Auditory CS	7.18	0.52	1.7	0.002
Experiment 2: Auditory CS	1.96	3.40	3.2	1.351
Experiment 3: Visual CS	1.85	2.71	3.4	1.288
Experiment 4: Somatosensory CS	22.52	0.21	0.5	0.001
All four experiments combined	5.94	0.75	1.7	0.002

*Note*. The parameters are of the best‐fitting gamma probability density function. Since we use a phenomenological approach, these parameters are not supposed to reflect biophysical reality. For example, the difference in onset latencies t_0_ between the response function of Experiment 1 and the response function of Experiment 2 can in part be explained by the fact that the best fitting gamma probability density function in Experiment 1 has a very shallow initial slope.

**Table 3:** Model Comparison

	AIC values (smaller is better)				*t*, *p*, and *d* values			
Experiment	Peak amplitude	AUC	GLM with response function from Experiment 1	GLM with response function from Experiment 1 plus derivative	Peak amplitude	AUC	GLM with response function from Experiment 1	GLM with response function from Experiment 1 plus derivative
Experiment 1: Auditory CS (*n* = 19)	‐40.5	‐36.6	‐46.2	‐53.5	*t*(18) = 4.41; *p* = .0003; *d* = 1.01	*t*(18) = 3.97; *p* = .0009; d = 0.91	*t*(18) = 5.05; *p* < 10^‐4;^ *d* = 1.16	*t*(18) = 5.89; *p* < 10^‐4^; *d* = 1.35
Experiment 2: Auditory CS (*n* = 12)	‐19.0	‐23.7	‐22.2	‐15.4	*t*(11) = 2.63; *p* = .0234; *d* = 0.76	*t*(11) = 3.29; *p* = .0073; *d* = 0.95	*t*(11) = 3.09; *p* = .0104; *d* = 0.89	*t*(11) = 2.10; *p* = .0593; *d* = 0.61
Experiment 3: Visual CS (*n* = 17)	‐24.0	‐20.1	‐25.9	‐25.5	*t*(16) = 2.71; *p* = .0155; *d* = 0.66	*t*(16) = 2.20; *p* = .0432; *d* = 0.53	*t*(16) = 2.96; *p* = .0093; *d* = 0.72	*t*(16) = 2.91; *p* = .0103; *d* = 0.71
Experiment 4: Somatosensory CS (*n* = 18)	‐22.9	‐23.4	‐28.5	‐26.1	*t*(17) = 2.46; *p* = .0248; *d* = 0.58	*t*(17) = 2.53; *p* = .0216; *d* = 0.60	*t*(17) = 3.16; *p* = .0058; *d* = 0.74	*t*(17) = 2.87; *p* = .0107; *d* = 0.68
Experiment 5: Long auditory CS (*n* = 15)	‐14.8	‐20.2	‐30.6	‐30.7	*t*(14) = 1.62; *p* = .1271; *d* = 0.42	*t*(14) = 2.43; *p* = .0289; *d* = 0.63	*t*(14) = 3.76; *p* = .0021; *d* = 0.97	*t*(14) = 3.78; *p* = .0020; *d* = 0.98

*Note*. Predictive validity with respect to differentiating CS+US‐ and CS‐ responses is given as Akaike Information Criterion (AIC; see Figure 2 for a graphical illustration of the same data). For completeness, *t*, *p*, and Cohen's *d* values with respect to differentiating CS+US‐ and CS‐ responses are listed for the two classic methods (peak scoring and comparing the AUC) and the two GLM‐based models. Two classic methods (peak scoring and comparing the AUC) are compared with the two GLM‐based models. The response function was developed on the basis of Experiment 1 (see Figure 1A).

### Model Validation in Auditory, Visual, and Somatosensory Fear Conditioning

Up to this point, we tested our model on the same data set that was used for model development. As this approach may be biased in favor of the model, we aimed at validating the model in independent data sets. To demonstrate wide generalizability, we used three fear conditioning experiments employing the same timing as Experiment 1 but three different sensory modalities of CS: auditory (as in the development data set), visual, and somatosensory CS (see Figure 1B–D for mean responses). In the second auditory experiment (Experiment 2), the model‐based approach was decisively better than peak scoring but did not differ from using the AUC. We note that this was the smallest data set because data from several participants had to be excluded due to missing values. In the visual experiment (Experiment 3), the model‐based approach provided better predictive validity than using the AUC to differentiate CS+US‐ and CS‐ but did not differ from using peak scoring. In the somatosensory experiment (Experiment 4), the model with the response function developed on the basis of the first auditory experiment decisively outperformed the two classic methods (see Figure 2 and Table 3 for indicators of predictive validity). In all three validation data sets, the model including the response function without its derivative provided better predictive validity than the model that included both the response function itself and its derivative. This difference between models with and without derivative was decisive in Experiment 2 but not in Experiment 3 and 4.

Taken together, our newly developed GLM‐based approach was better than or at least as good as the two commonly used approaches across three sensory modalities of CS. This result holds even though pupil responses to the CS differed in shape across the four experiments.

For illustration, we derived response functions for the three validation data sets separately and additionally a response function based on the first four experiments combined (Figure 1B–E, see Table 2 for parameter estimates). This latter response is based on the maximum amount of data (with the same timing) available to us, and thus we cannot validate it using independent data. Incorporating this response function in our model resulted in values of predictive validity that allowed similar conclusions to those derived from the a priori model development data set (see Table 4). We suggest using—and thus validating—this response function for analyzing future fear conditioning experiments.

**Table 4:** Model Comparison with a Response Function Based on the First Four Experiments

	AIC values (smaller is better)		*t*‐, *p*‐ and *d*‐values	
Experiment	GLM with response function from first four experiments	GLM with response function from first four experiments plus derivative	GLM with response function from first four experiments	GLM with response function from first four experiments plus derivative
Experiment 1: Auditory CS (n = 19)	‐41.6	‐51.4	*t*(18) = 4.54; *p* = .0003; *d* = 1.04	*t*(18) = 5.64; *p* < 10^‐4^; *d* = 1.29
Experiment 2: Auditory CS (*n* = 12)	‐23.8	‐14.1	*t*(11) = 3.30; *p* = .0070; *d* = 0.95	*t*(11) = 1.90; *p* = .0831; *d* = 0.55
Experiment 3: Visual CS (*n* = 17)	‐25.1	‐25.5	*t*(16) = 2.85; *p* = .0116; *d* = 0.69	*t*(16) = 2.90; *p* = .0105; *d* = 0.70
Experiment 4: Somatosensory CS (*n* = 18)	‐27.8	‐27.0	*t*(17) = 3.07; *p* = .0069; *d* = 0.73	*t*(17) = 3.00; *p* = .0085; *d* = 0.70
Experiment 5: Long auditory CS (*n* = 15)	‐28.2	‐26.0	*t*(14) = 3.47; *p* = .0037; *d* = 0.90	*t*(14) = 3.19; *p* = .0066; *d* = 0.82

*Note*. In contrast to Table 3, here the first four experiments were combined to derive the response function used for estimating the values presented in this table (see Figure 1E). Predictive validity with respect to differentiating CS+US‐ and CS‐ responses is given as Akaike Information Criterion (AIC). For completeness, *t*, *p*, and Cohen's *d* values are given.

### Model Validation in Auditory Fear Conditioning with a Longer CS Presentation

**Figure 3:**
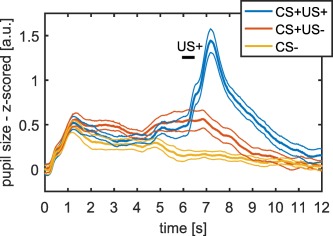
Mean pupil size responses (left) for auditory fear conditioning with long CS presentation (Experiment 5). CS presentation lasted from 0 s to 6.5 s and US delivery occurred at 6 s after CS onset. Thick lines represent mean responses and thin lines represent *SEM*.

We validated our model in an additional independent data set, in which the CS presentation lasted 6.5 s instead of 4 s (using the response function from Experiment 1 and the response function from the first four experiments combined). Our model‐based approach outperformed the two classic methods (see Figure 2 and Table 3 and 4 for model comparison and Figure 3 for mean responses).

### Comparing Pupil Model with Skin Conductance and Heart Period Models

To put our results into a psychophysiological context, we sought to compare them to other peripheral measurement modalities often used to assess fear conditioning. We have previously developed PsPM approaches for analyzing fear conditioned SCR and heart period responses (Castegnetti et al., 2016; Staib et al., 2015). Therefore, we aimed at comparing these three measurement modalities (see Table 5 for indicators of predictive validity).

**Table 5:** Comparison of Measurement Modalities

	AIC values (smaller is better)			*t* and *p* values		
Tasks	Pupil (GLM with response function from Experiment 1)	SCR	Heart period	Pupil (GLM with response function from Experiment 1)	SCR	Heart period
Experiment 1: Auditory CS (*n* = 13)	−24.6	−15.7	−20.3	*t*(12) = 3.27; *p* = .0067	*t*(12) = 2.03; *p* = .0648	*t*(12) = 2.69; *p* = .0196
Experiment 2: Auditory CS (*n* = 10)	−15.2	−16.9	−18.7	*t*(9) = 2.33; *p* = .0447	*t*(9) = 2.59; *p* = .0290	*t*(9) = 2.87; *p* = .0184
Experiment 3: Visual CS (*n* = 17)	−25.9	−15.8	NA	*t*(16) = 2.96; *p* = .0093	*t*(16) = 1.54; *p* = .1431	NA
Experiment 4 Somatosensory CS (*n* = 18)	−28.5	−33.7	NA	*t*(17) = 3.16; *p* = .0058	*t*(17) = 3.76; *p* = .0016	NA

*Note*. Results are based on the maximum available number of participants for the respective measurement modalities. Predictive validity with respect to differentiating CS+US‐ and CS‐ responses is given as Akaike Information Criterion (AIC). For completeness, *t* and *p* values with respect to differentiating CS+US‐ and CS‐ responses are listed for all three measurement modalities. The response function for the pupil model was developed from data of Experiment 1 (see Figure 1A).

The pupil model provided decisively better predictive validity than the SCR and heart period models in Experiment 1. In Experiment 2, the pupil did not differ from the SCR model but was decisively worse compared with the heart period model. In Experiment 3 and 4, we recorded SCR but not heart period data. In Experiment 3, the pupil model outperformed the SCR model, while in Experiment 4 the opposite was the case. To summarize, the pupil response model decisively outperformed the SCR model in two out of four experiments, performed better than the heart period model in one experiment, and decisively worse in another.

To explore whether a common underlying neural process drives the three different autonomic responses, we correlated the differences between estimated response amplitudes for CS+US‐ and CS‐ trials across participants. In Experiment 1, pupil measurements were correlated to SCR and to heart period measurements—pupil and SCR: Pearson's *r* = .741; *p* = .0038; pupil and heart period: *r* = .717; *p* = .0058; SCR and heart period: *r* = .656; *p* = .0149; across all four experiments, we tested eight correlations and therefore the appropriate Bonferroni‐corrected significance level was .0063. In Experiment 2, none of the correlations survived Bonferroni correction—pupil and SCR: *r* = .157; *p* = .6639; pupil and heart period: *r* = .731; *p* = .0164; SCR and heart period: *r* = .481; *p* = .1598. In Experiment 3 and 4, only pupil and SCR were measured. After Bonferroni‐correction, the two measures did not correlate significantly in Experiment 3, *r* = .477; *p* = .0528, and showed a trend in Experiment 4, *r* = .603; *p* = .0080. Overall, we tentatively infer from these correlation analyses that pupil, SCR, and heart period models capture some degree of common variance, which might suggest a common underlying neural process. But further experiments directly aimed at testing this are needed.

In sum, comparing the newly developed pupil response model to state‐of‐the‐art analyses of SCR and heart period responses showed that the best measurement modality depended on the data set and thus possibly on the sensory modality of the CS. Hence, our current findings do not establish an overall superiority of any of the three measurement modalities. Nevertheless, our results convey that analyzing fear‐conditioned pupil size responses is a robust and useful alternative to SCR and heart period responses.

### Additional Pupil Model Including US Response

**Figure 4:**
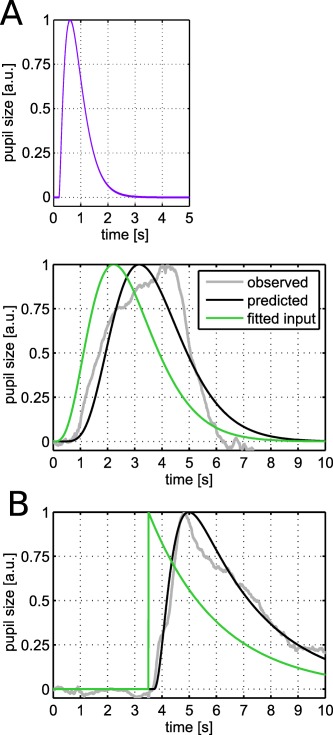
Fitted input into the pupillary system elicited by (A) the CS+US‐ and (B) the US+. The observed data is taken from Experiment 1 (see Figure 1A). The inset shows in purple the luminance‐related response function developed in our previous study (Korn & Bach, 2016). Convolving the fitted input with the luminance‐related pupillary response function results in the predicted output of the system. A: Observed data are the difference between the means for CS+US‐ and CS‐ trials. B: Observed data are the difference between the means for CS+US+ and CS+US‐ trials.

Up to this point, we did not explicitly estimate responses in CS+US+ trials in the GLMs, since we wanted to exclude the possibility that US+ responses may contaminate the estimation of the CS+ versus CS‐ difference. However, including an explicit model of the response to the US+ could mitigate this problem and may allow for increasing the amount of trials by using both CS+US‐ and CS+US+. This might offer a better discrimination of responses to CS+ versus CS‐. We therefore derived a response function for the response to the US+ on the basis of the mean difference of CS+US+ and CS+US‐ trials from Experiment 1. Parameters for the function of the response to the US+ were *k* = 1.90; *θ* = 1.57 s^−1^; *c* = 2.9; *t*_0_ = 3.76 s; for comparability, the onset defined with respect to the beginning of the CS (i.e., 3.5 s before the US onset). The two response functions for US+ and CS+ shared only a small amount of variance in the total 10‐s window considered (*R^2^* = .0218; see also the two predicted responses in Figure 4A,B), such that their input amplitudes could independently be estimated in a GLM.

We then implemented these two response functions in a model that included an intercept and five additional regressors (one regressor for all CS+ responses, one for all CS‐ responses, one for US+ responses, one for US‐ responses in CS+US‐ trials, one for US‐ responses in CS‐ trials). In Experiment 1, 3, and 4, this model allowed a decisively better predictive validity for discriminating CS+ and CS‐ responses than the model that only takes CS+US‐ trials into account, and was equal in Experiment 2 (compare Table 3 with Table 6).

**Table 6:** Model Accounting for Responses to the US

Experiment	AIC values (smaller is better)	*t* and *p* values
Experiment 1: Auditory CS (*n* = 19)	−54.2	*t*(18) = 5.97; *p* < 10^−4^
Experiment 2: Auditory CS (*n* = 12)	−29.9	*t*(11) = 4.15; *p* = .0016
Experiment 3: Visual CS (*n* = 17)	−29.0	*t*(16) = 3.33; *p* = .0043
Experiment 4: Somatosensory CS (*n* = 18)	−33.3	*t*(17) = 3.72; *p* = .0017

*Note*. Predictive validity with respect to differentiating CS+ and CS‐ responses in model that explicitly accounts for (potential) responses to the US in CS+US+ and CS+US‐ trials. Akaike Information Criterion (AIC) as well as *t* and *p* values are listed. A comparison with the values given in Table 3 shows that the model that includes response functions for responses to the US performs better for all experiments.

In addition, this model has the potential to address whether the omission of the US+ in CS+US‐ trials elicits a response that resembles the response to the US+ itself (Bach & Friston, 2012; Spoormaker et al., 2012). We did not find strong evidence for such an overall omission response: Parameter estimate for US‐ in CS+US‐ trials did not differ from parameter estimates for US‐ in CS‐ trials in Experiment 1, 3, and 4. The only significant difference emerged in Experiment 2, *t*(11) = 4.35; *p* = .0046, after Bonferroni correction for multiple testing. In contrast, the parameter estimates for responses to the US+ were significantly different from zero and from the parameter estimates for US‐ trials, all *p*s < .01 after Bonferroni correction.

To make sure that the CS+ estimation in the CS+US+ trials was not biased by US+ occurrence, we tested an additional model with two separate regressors for responses to the CS+ in CS+US+ and CS+US‐ trials. The parameter estimates for the CS+US+ and CS+US‐ regressors did not differ significantly from each other in any of the experiments (after Bonferroni correction).

Thus, a model that explicitly accounts for US responses may have the potential to further improve the CS+ and CS‐ discrimination based on pupillary responses, but some caution is warranted to ensure that the CS estimation in CS+US+ trials is not biased by the US+ response.

### Estimating the Inputs into the Pupillary System

Intriguingly, defining response functions for pupil responses during fear conditioning can serve two purposes: First, they can be used for specifying PsPM approaches to distinguish CS+ and CS‐ trials based on pupil time series, as demonstrated above. Second, they can provide information about the time course of the upstream signals impinging on the pupillary system. We have previously characterized similar inputs in three different tasks (an auditory oddball task, an emotional word task, and a perceptual discrimination task; Korn & Bach, 2016). Here, we provide an analogous characterization with respect to fear conditioning.

Under the assumption that pupis responses (PSR) elicited by fear conditioning paradigms share a common final pathway with PSR elicited by luminance changes, we can estimate the inputs into this final pathway. We used our previous characterization of luminance‐related PSR (Korn & Bach, 2016) and fitted inputs (with the form of a gamma probability distribution) so that the predicted output of the pupillary system approximated the observed pupil time series for the response to CS+US‐ (Figure 4A) and US+ (Figure 4B). As expected, the fitted inputs for these two responses differed markedly in shape: The input into the response to the US+ was much steeper and peaked rather immediately at US+ onset (i.e., at 3.5 s). In contrast, the input into the response to the CS+US‐response was shallower and peaked around 2 s after CS+ onset.

## Discussion

In this article, we present a PsPM that allows assessing fear learning from pupil time series. This model can be readily used in experimental practice. Importantly, we showed that stimuli of three different sensory modalities led to similar fear‐conditioned pupil size responses. Our PsPM allowed decisively better or equal discrimination of CS+ and CS‐ than the two methods commonly used in the literature (i.e., the model showed higher or equal predictive validity). This was found not only in the data set on which the response function was developed but also in four independent data sets with auditory, visual, and somatosensory CS. The relative performance of the two extant methods differed between the validation data sets without an obvious pattern, something that urges further investigation. However, when regarded across all experiments, our PsPM approach provided the best predictive validity. Modeling the derivative of the response function in addition to the canonical response function itself reduced predictive validity in the validation data sets such that a model containing only the canonical response function emerged as best option.

Our aim was not to provide a model physiological realism, and the formalization of the response function in terms of a gamma probability density function was based on mathematical parsimony. Instead, our model fulfilled the aim of reliably distinguishing between CS+ and CS‐ trials. This was the case, although mean pupillary responses differed between sensory modalities. Incorporating modality‐specific differences may enhance predictive validity within the same modality at a loss of generality. We finally formulate a response function that is based on all four experiments with the same timing and thus on the maximum amount of data available to us. This response function must be validated in the future using independent data sets. Our response functions were based on the differences between the mean responses to CS+US‐ and CS‐ responses. We have previously shown for heart period responses and respiratory responses that there is no theoretical reason and indeed no empirical evidence that this approach overestimates CS+/CS‐ differences (Castegnetti et al., 2016; Castegnetti et al., in press).

Our model provides a powerful approach to assess the question whether fear conditioning can be inferred from pupil size responses or not in a given data set. The model is thus not aimed at characterizing the forward relation from psychological processes to pupil size but instead at making the reverse statement about the most likely psychological process given the data. Put differently, a decoding classification (Ghaderyan & Abbas, 2016; Kriegeskorte, 2011; Naselaris, Kay, Nishimoto, & Gallant, 2011; Poldrack, 2011) was not the goal of the study since the experimenter sets a priori when CS+ and CS‐ occur within the experiment. Instead, the model is optimized for features of the pupil response traces that are indicative of the learning process in fear conditioning. This makes our model useful for researchers who want to probe for experimental manipulations or for participant characteristics that could potentially alter the underlying learning process (e.g., fear extinction, social context, or phobic patients; Kroes, Schiller, LeDoux, & Phelps, 2016). Indeed, by providing equal or higher predictive validity than peak scoring or calculating the AUC, our model increases statistical power, which is beneficial for analyzing such experimental manipulations.

Four of the experiments reported here employed a delay fear conditioning paradigm with the same CS/US interval of 3.5 s and the same CS/US contingencies (i.e., the CS+ predicted the US in 50% of the cases). In an additional experiment with longer CS presentation, the model outperformed classic methods and therefore may very well generalize to different timings. Nevertheless, it is an open question how well the model generalizes to delay conditioning paradigms with different contingencies, to trace conditioning paradigms, and/or to reward conditioning paradigms. We expect that the same model will be useful in experiments that vary, for example, CS/US contingencies or US strength. We also expect our model to be applicable for different timings. The shape of the inferred response function depends in part on the fact that the pupil showed a sustained dilation for CS+ trials that was reduced or absent in CS‐ trials. A longer interval between CS and US onsets seems to extend this prolonged dilation (Reinhard et al., 2006). Therefore, the same response function may still confer good predictive validity, although even better results might be obtained from a specifically developed response function that captures such a prolonged dilation.

Interestingly, a study using reward conditioning in monkeys showed that lesions of the anterior cingulate cortex reduced the sustained dilation typically observed in CS+ trials (Rudebeck et al., 2014) while initial responses to the CS were preserved. This suggests that different neural mechanisms mediate temporally separable components of conditioned pupil responses. Our model can be easily extended to allow for dissociating such components. We provided a first step toward this goal by proposing an additional model that explicitly incorporated a separate response function for the pupil responses to the US. This model possessed the added advantage that both CS+US‐ and CS+US+ trials could be used for differentiating CS+ and CS‐ trials.

We tested whether the pupil allowed a better characterization of fear conditioning than two other physiological measurement modalities commonly used for this purpose: SCR and heart period responses (Castegnetti et al., 2016; Staib et al., 2015). The pupil response model outperformed the SCR model in two out of four data sets and the heart period model in one out of two data sets. Thus, these results were not entirely conclusive. It is an open empirical question whether the best measurement methods—and thus PsPM analysis approaches—depend on the sensory modality of the CS or whether they depend on differences between participant samples. At the very least, our findings show that the pupil provides a reliable measure of fear conditioning that can improve or complement other measurement modalities. We also found initial and tentative evidence that the indicators for fear conditioning obtained from pupil size, SCR, and heart period responses correlated with each other to some degree, which suggests that these capture a shared underlying neural process. Such correlations are interesting from the vantage point of the underlying neuroanatomy. SCR are solely determined by the sympathetic branch of the autonomous nervous system and heart period responses—at least, the early responses—are influenced by the parasympathetic branch (Paulus et al., 2016). In contrast, pupil size is antagonistically related to both sympathetic and parasympathetic branches for constriction and dilation, respectively (McDougal & Gamlin, 2008). Thus, pupil responses can be expected to correlate with both SCR and heart period responses, which is what we found—at least in Experiment 1. A promising avenue for future research would be to assess possibly differential correlations between these measurement modalities across different fear conditioning paradigms.

We have previously proposed a PsPM to characterize how the pupil reacts to luminance changes (Korn & Bach, 2016). We harnessed this model to infer the likely time course of the inputs of the central nervous system into the pupillary system that elicit the fear‐conditioned pupil responses, under the assumption of a common final pathway in the peripheral pupillary system. We obtained the plausible result that the inputs eliciting US+ responses are more peaked and rapid than those eliciting the difference between CS+ and CS‐ responses. It will be interesting to extend these analyses to setups with different timings and possibly different contingencies between CS and US. This may offer a way to reliably infer whether the pupil responses, which allow the differentiation of CS+ and CS‐, are locked in time to the occurrence of the CS or whether they in part depend on the timing of the US.

One key advantage of fear conditioning paradigms rests in their applicability across different species. Nevertheless, popular readouts such as SCR or freezing are applicable mainly in humans and rodents, respectively. Pupillary responses may offer a common measurement modality across species. This is especially pertinent since recent investigations in rodents (McGinley, David, & McCormick, 2015; Reimer et al., 2014), monkeys (Ebitz & Platt, 2015; Joshi, Li, Kalwani, & Gold, 2016; Rudebeck et al., 2014), and humans (Eldar, Cohen, & Niv, 2013; Yellin, Berkovich‐Ohana, & Malach, 2015) have elucidated the relationship between pupil size and neural signals from cortical and subcortical structures. The basic logic of our GLM‐based approach can be readily extended to nonhuman pupil responses. An intriguing possibility is that the response function derived here will generalize to other mammals.

In sum, we provide an explicit psychophysiological model for pupillary responses that allows a reliable characterization of human fear conditioning, and can thus complement or supersede existing measures.
